# In situ detection of ferric reductase activity in the intestinal lumen of an insect

**DOI:** 10.1007/s00775-024-02080-y

**Published:** 2024-12-01

**Authors:** Anna Karen Hernández-Gallardo, Trinidad Arcos-López, Jahir Marceliano Bahena-Lopez, Carlos Tejeda-Guzmán, Salvador Gallardo-Hernández, Samuel M. Webb, Thomas Kroll, Pier Lorenzo Solari, Carolina Sánchez-López, Christophe Den Auwer, Liliana Quintanar, Fanis Missirlis

**Affiliations:** 1 Departamento de Fisiología, Biofísica y Neurociencias, Cinvestav, 07360 Mexico City, Mexico; 2 Departamento de Química, Cinvestav, 07360 Mexico City, Mexico; 3 Departamento de Física, Cinvestav, 07360 Mexico City, Mexico; 4 Stanford Synchrotron Radiation Lightsource, SLAC National Accelerator Laboratory, Menlo Park, CA 94025 USA; 5 Synchrotron Soleil, L’Orme des Merisiers, Départementale 128, 91190 Saint-Aubin, France; 6 Centro de Investigación sobre el Envejecimiento, Cinvestav, 14330 Mexico City, Mexico; 7 Université Côte d’Azur, CNRS, Institut de Chimie de Nice, 06108 Nice, France

**Keywords:** EPR, Iron uptake, Malvolio, Microscopy, Synchrotron, XANES, X-ray fluorescence imaging

## Abstract

The rise of atmospheric oxygen as a result of photosynthesis in cyanobacteria and chloroplasts has transformed most environmental iron into the ferric state. In contrast, cells within organisms maintain a reducing internal milieu and utilize predominantly ferrous iron. Ferric reductases are enzymes that transfer electrons to ferric ions, either extracellularly or within endocytic vesicles, enabling cellular ferrous iron uptake through Divalent Metal Transporter 1. In mammals, duodenal cytochrome b is a ferric reductase of the intestinal epithelium, but how insects reduce and absorb dietary iron remains unknown. Here we provide indirect evidence of extracellular ferric reductase activity in a small subset of *Drosophila melanogaster* intestinal epithelial cells, positioned at the neck of the midgut’s anterior region. Dietary-supplemented bathophenanthroline sulphate (BPS) captures locally generated ferrous iron and precipitates into pink granules, whose chemical identity was probed combining in situ X-ray absorption near edge structure and electron paramagnetic resonance spectroscopies. An increased presence of manganese ions upon BPS feeding was also found. Control animals were fed with ferric ammonium citrate, which is accumulated into ferritin iron in distinct intestinal subregions suggesting iron trafficking between different cells inside the animal. Spectroscopic signals from the biological samples were compared to purified *Drosophila* and horse spleen ferritin and to chemically synthesized BPS-iron and BPS-manganese complexes. The results corroborated the presence of BPS-iron in a newly identified ferric iron reductase region of the intestine, which we propose constitutes the major site of iron absorption in this organism.

**Graphical abstract:**

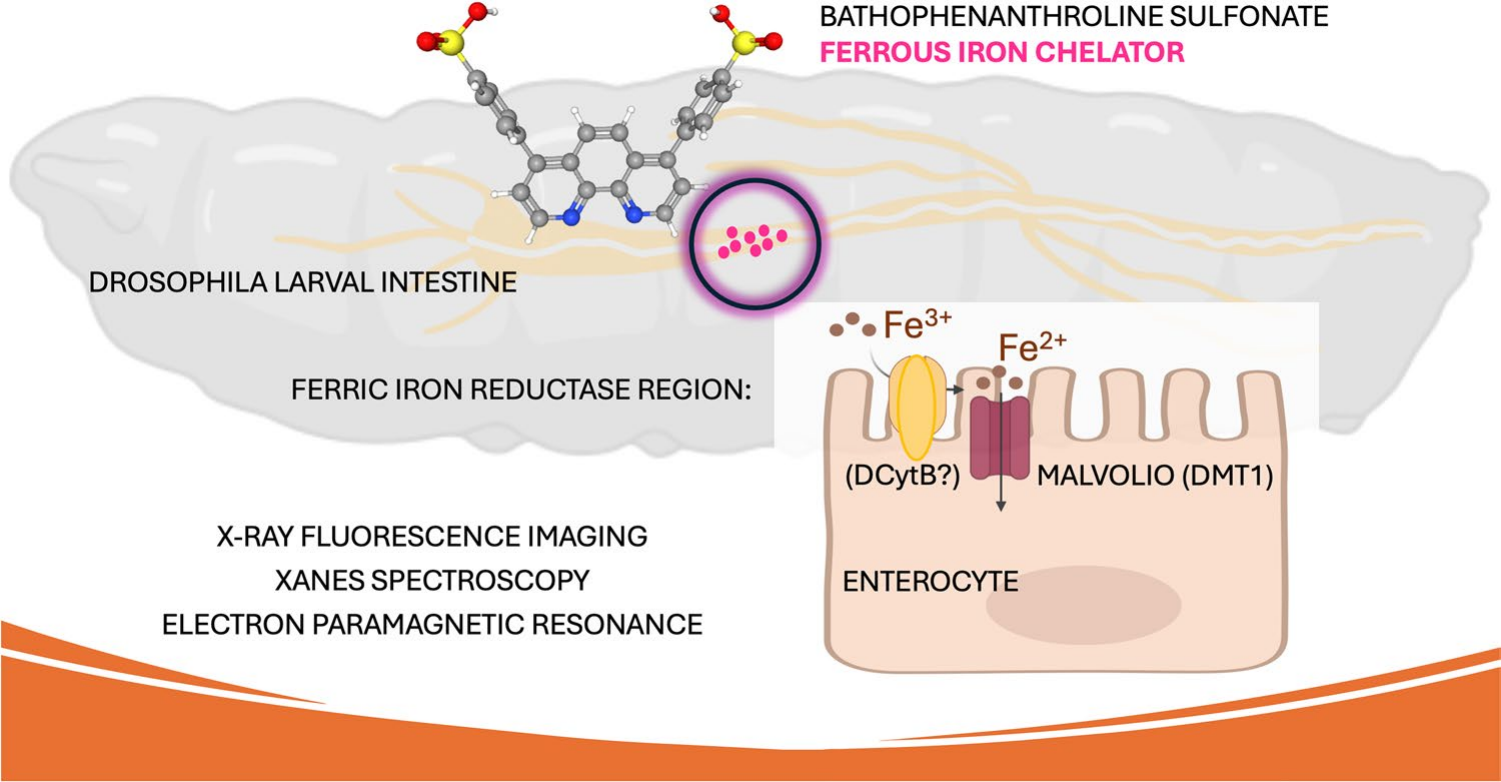

**Supplementary Information:**

The online version contains supplementary material available at 10.1007/s00775-024-02080-y.

## Introduction

Iron is an essential nutrient for all animals due to its presence as a cofactor in multiple enzymes [1–3], where iron ions bind directly to the active sites of proteins, or in the form of iron-sulfur clusters, or as the core of protoporphyrin in heme [4–6]. For this reason and because of the crucial role of iron for hemoglobin synthesis [7, 8] and therefore peripheral oxygen transport [9, 10], iron deficiency is a major public health concern in humans [11–13]. Intestinal iron absorption has received particular attention (reviewed in [14–17]) and the mechanisms of iron absorption have been investigated in only a handful of the approximately one million insect species that colonize diverse microenvironments on the planet. Despite variations in theme, key molecular components involved in iron metabolism were found to be evolutionarily conserved between insects and mammals [18–22].

The primary route of entry for iron into intestinal epithelial cells is through the Divalent Metal Transporter 1 (DMT1), as evidenced by the anemia resulting from mutations in the respective homologous genes from mouse [23], rat [24], zebrafish [25] and human [26–28]. The insect gene homologous to DMT1 has been termed *Malvolio* based on the homonymous character in William Shakespeare's comedy “Twelfth Night, or What You Will” whose “distempered appetite” affected his taste; mutant *Drosophila melanogaster* flies for DMT1/Malvolio failed to discriminate sucrose-supplemented from sugar-free diets for reasons that remain unclear [29]. Loss of *Malvolio* function was later shown to cause iron deficiency in flies [30] and mosquitoes [31], while the gene is transcriptionally regulated in response to iron [32]. Thus, divalent ferrous iron is the bioavailable ionic form of iron in both insect and vertebrate intestinal epithelia.

Although the speciation and redox state of iron in animal diets is not established [33, 34], ambient oxygen makes it likely that a significant portion of the iron is present in the ferric form [35]. This inference is supported by the almost ubiquitous chemical preference of bacterial siderophores to chelate ferric iron ions in the quest of bacteria to capture iron from the environment [36]. From these considerations, it follows that intestinal epithelia require ferric reductase activity to convert ferric to ferrous iron in the vicinity of DMT1 on the cell membrane [37–40]. Indeed, at least one iron-regulated ferric reductase, Duodenal cytochrome b (Dcytb), has been identified in mice [41] and shown to support DMT1-dependent iron transport in human cell culture [42, 43], although the Dcytb knockout mice under normoxic conditions appeared to compensate for the lack of this protein [44, 45], suggesting the potential presence of dietary sources of reducing agents, such as ascorbate (vitamin C) [46, 47], or of other ferric reductases that remain to be identified.

Indeed, a variety of ferric iron reductases have been described in the microbial world, in yeasts and in plants, even prior to their identification in animals (reviewed in [48–51]). In contrast, no experimental results with ferric reductases of insects are present in the scientific literature; however, a recent article presented a bioinformatics-based analysis of Cytb561 family members from nine species representing eight insect orders summarizes potential candidate genes and their phylogenetic relationships [52]. While feeding *Drosophila* larvae with bathophenanthroline sulfate (BPS), a non-membrane-permeable ferrous iron chelator that efficiently prevents iron absorption upon dietary administration [53–59], we noticed a pink precipitate on the apical side of the intestinal epithelium. In this paper, we characterize the precipitate by in situ X-ray absorption spectroscopy (XAS) and electron paramagnetic resonance (EPR), comparing the results to spectra from BPS–ferrous complex synthesized under aerobic conditions and to ferritin spectra, where iron from the ferric hydroxide core of the protein nanocage predominates. In accordance with prior examples of the use of BPS as a marker [60, 61], we propose that the staining identifies a physiological site of insect ferric reductase activity that should correspond to an entry point of divalent ferrous iron through the Malvolio transporter into the intestinal epithelium.

## Materials and methods

### Animals

Isogenic *D. melanogaster* flies that carry no known mutations were previously established in our laboratory and referred to as *w*^+^ [62]. They were maintained at 25 °C on standard media based on yeast and molasses. Wandering third instar larvae were used for all dissections of intestines, whereas adult flies of mixed sex were used to purify *Drosophila* ferritin.

### Prussian blue staining

Wandering third instar *w*^+^ larvae grown in an iron rich diet supplemented with 1 mM ferric ammonium citrate (FAC), or in an iron poor diet supplemented with 250 µM BPS. BPS is a membrane-impermeable iron chelator that effectively diminishes iron absorption [53–59]. The larvae were washed in phosphate-saline buffer (PBS) three times for 5 min prior to dissections. Intestines were dissected in PBS and fixed with 4% formaldehyde in PBS for 20 min at room temperature, then washed three times for 15 min with 0.3% Triton–PBS. Next, samples were incubated at room temperature for 1 h in fresh staining solution (2% K_4_Fe(CN)_6_ + 2% HCl) protected from the light and then washed three times with PBS to remove any remaining staining solution. Images were taken using a Leica EZ4 HD Stereo Microscope on either white or black background.

### Ferritin isolation protocol

20 g of flies raised as larvae on diet containing 1 mM FAC were collected in liquid nitrogen and pulverized in a mortar under liquid nitrogen. The pulverized flies were suspended in a freshly prepared 5 mM phenylthiourea (Cat No. 103-85-5) and 50 mM HEPES, pH 7.5, solution (Cat No. 7365-45-9) with the use of a Dounce homogenizer. The fly homogenate was sonicated 5 times for 45 s at 55% maximum power on a Sonics Vibra Cell at 40 °C. The suspension was centrifuged at 15,000*g* for 15 min at 40 °C in a Thermo Scientific Sorvall Legend Micro 21 R centrifuge. After discarding the top fat layer, the supernatant was filtered through four layers of Whatman (Cat No. 1001 125) filter paper. The filtrate was collected and evenly distributed in thick wall polyallomer ultracentrifuge tubes and then submitted to ultracentrifugation at 180,000*g* (Beckman 75 Ti rotor) for 1 h at 15˚C. The resulting sticky brownish pellet containing ferritin was collected from the tubes and then resuspended with 50 mM HEPES pH 7.5. Vortex was used to break down big chunks of pellet. After complete pellet resuspension the sample was incubated at 70 °C for 15 min following a centrifugation step at 15,000 g for 15 min at 40 °C. A saturated KBr (Cas No. 7758-02-3) solution was prepared as follows: KBr was added to a HEPES 50 mM solution until a white precipitate was observed. This saturated KBr solution was placed in thick wall polyallomer ultracentrifuge tubes and then the suspended pellet was placed on top of it and layers were observed. A final ultracentrifugation step in a saturated KBr solution at 180,000*g* for 22 h at 15 °C resulted in a sticky brownish sample of ferritin. The pellet was retrieved and resuspended in 50 mM HEPES, pH 7.5. Excess KBr was removed by ultrafiltration using Viovaspin 500 (10 kDa MWCO) columns.

### BPS–Fe^2+^ synthesis

A BPS–Fe^2+^ solution was prepared in aerobic conditions using Bathophenanthrolinedisulfonic acid disodium salt hydrate (BPS) from Sigma Aldrich (Cat No. 527-46-49-3) and 7-hydrated ferrous sulphate from Baker (Cat No. 7782-63-0). BPS is a membrane-impermeable chelator that binds ferrous iron with a ratio of three chelator molecules to one metal ion [63–65]. Hence, final concentrations were adjusted to BPS 450 mM and Fe^2+^ 150 mM.

### X-ray fluorescence imaging and spectroscopy

The midgut portion of the larval intestines was dissected from 3rd instar wandering larvae (grown on diets supplemented either with 1 mM FAC or with 250 µM BPS) in a 0.25 M sucrose solution and placed on microscope slide coverslips (Thermo Scientific Nunc Thermanox) to air-dry at room temperature. These were imaged with a Leica EZ4 HD Stereo Microscope and, subsequently, X-ray fluorescence images were collected at the Stanford Synchrotron Radiation Lightsource (SSRL) using beam line 2–3. The measurements were conducted with the Stanford Positron Electron Accelerating Ring storage ring containing 500 mA in top-off mode at 3.0 GeV. The incident X-ray beam was obtained using a Si (111) double-crystal monochromator to achieve the energy of choice. For Fe X-ray Absorption Near Edge Spectroscopy (XANES), the energy of the monochomator was calibrated to the first inflection point of an Fe metal foil set to 7112 eV. The fluorescence emission line of iron, as well as the intensity of the total scattered X-rays, were monitored using a silicon drift Vortex detector (SII NanoTechnology) mounted at 90 deg to the incident beam. Photon processing was accomplished with Xpress3 signal processing electronics (Quantum Detectors). An Rh-coated Kirkpatrick–Baez mirror pair (Xradia) provided a microfocused beam of 3 × 3 microns. The incident and transmitted X-ray intensities were measured with nitrogen-filled ion chambers. Samples were mounted at 45 deg to the incident X-ray beam and were spatially rastered in the microbeam using a Newport VP-25XA-XYZ stage. Sample exposure was 100 ms per pixel. Fluorescence signals were normalized against the incident X-ray beam intensity to normalize against its fluctuations. Data analysis was performed using the MicroAnalysis Toolkit computer program [66]. No smoothing or related data manipulations were performed.

XAS measurements were also performed for control substances on the MARS beamline of the SOLEIL synchrotron facility [67]. The main optics of the beamline consist essentially of a water-cooled double-crystal monochromator (FMB Oxford) which is used to select the incident energy of the X-ray beam. For horizontal focusing, and two large water-cooled reflecting mirrors (IRELEC/SESO) that are used for high-energy rejection (harmonic part) and vertical collimation and focusing. For this study, the monochromator was set with Si(111) crystals and the mirrors were set with Si reflecting stripes at an angle of 3.1 mrad. The measurements were performed in fluorescence mode at the Fe K edge (calibrated to Fe metal at 7112 eV), to collect the ray Kα of the Fe fluorescence line (6403.84 eV). The fluorescence signal was collected using a 4-element silicon drift detector (HITACHI) and an additional mono silicon drift (HITACHI).

### Electron paramagnetic resonance

All EPR samples were prepared in 50 mM 4-hydroxyethylpiperazineethanesulfonic acid (HEPES) buffer with 50% glycerol as cryoprotectant. Commercially available horse spleen ferritin (HSF) samples were prepared at a concentration range of 2.5–3.75 mg/mL. *Drosophila* ferritin (DF) was purified from adult flies that were raised on a 1 mM FAC diet, and samples for EPR were 6 mg/mL. The BPS–Fe complex was prepared as described above. Gut–FAC and gut–BPS samples for EPR analysis were prepared from 60 larva intestines of *D. melanogaster* that were raised on diets supplemented with 1 mM FAC or 250 µM BPS, respectively. X-band (9.4 GHz) EPR spectra were collected using an EMX Plus Bruker System, with an ER 041 XG microwave bridge and an ER 4102ST cavity. EPR spectra were recorded at 150 K using an ER4131VT variable temperature nitrogen system. The following conditions were used: microwave power, 10 mW; modulation amplitude, 5 G; modulation frequency, 100 kHz; time constant, 327 ms; conversion time, 82 ms, a field sweep was made from 0 to 5000 G, with 4092 data points, averaging over 3 scans. EPR spectra were baseline-corrected using KaleidaGraph 5.01.

### Inductively coupled plasma optic emission spectrometry

For metal concentration determinations, twenty wandering third instar larvae were digested in 1 mL concentrated Nitric Acid (Metal-Free, Suprapur) at 200 °C using the MARS6 microwave digestion system (CEM Corporation). Inductively Coupled Plasma Optical Emission Spectroscopy (ICP-OES) was performed on a PerkinElmer Optima 8300 instrument (CT, USA) and metal concentrations were determined using calibration curves of standard solutions [68].

## Results

**Fig. 1:**
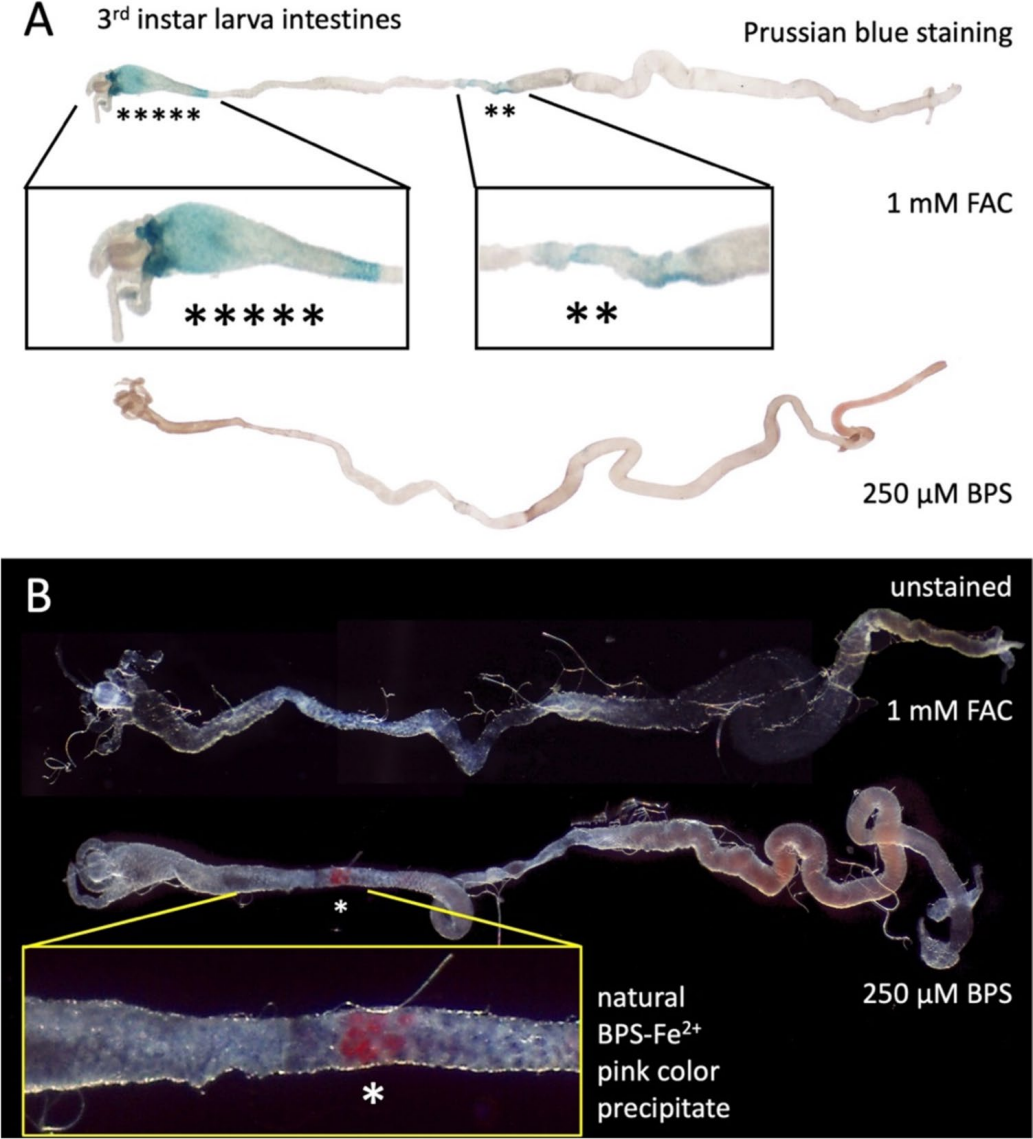
A pink BPS–Fe^2+^ precipitate is observable in a small distinct region of the anterior midgut. **A** Prussian blue staining of intestines dissected out of wandering 3rd instar larvae of *Drosophila melanogaster* that were raised on diets supplemented with 1 mM FAC (top) or 250 µM BPS (bottom). Insets show close-ups of stained regions (blue color). Five asterisks depict the iron-inducible anterior region (left panel), two asterisks depict the “iron region” that typically accumulates iron from basal media [30, 32, 53, 69]. Note that there is no blue precipitate along the entire intestinal tract when larvae were grown in the presence of BPS. **B** Freshly dissected intestines as above but unstained. Asterisk denotes a characteristic pink color precipitate only in the animals that consumed BPS-supplemented diet (magnified in the inset). Anterior is always to the left

Iron supplementation of a typical laboratory diet for *D. melanogaster* with 1 mM FAC leads to evident iron accumulation in the so-called “iron region” in the middle midgut and in the anterior midgut, both readily detectable with Prussian Blue staining (Fig. 1A). In contrast, dietary supplementation with 250 µM BPS depletes iron from the entire intestinal epithelium [53] (Fig. 1A, bottom intestine). As expected [30, 53–55], these treatments also result in 163% increase or 50% decrease in total body iron, respectively (Fig. S1A, SI). We observed a pink precipitate forming at the “neck” of the anterior intestine in BPS-treated larvae. The precipitate appeared posteriorly adjacent to the region that accumulates ferritin iron in FAC-treated larvae (Fig. 1B).

**Fig. 2:**
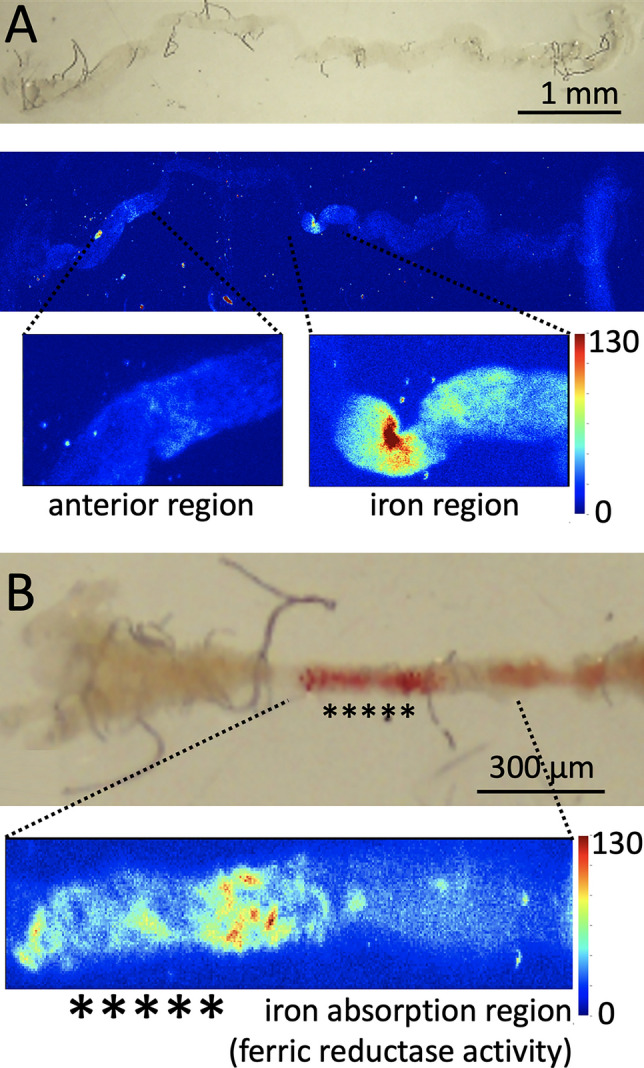
X-ray fluorescence imaging of air-dried intestines dissected out of *Drosophila melanogaster* 3rd instar larvae. The X-ray fluorescence images are from the same samples photographed. **A** Sample from a larva raised on 1 mM FAC. Top panel shows an image of the entire midgut taken at the stereoscope. Middle panel shows an X-ray fluorescence image of iron at the same scale. Two regions accumulate iron and have been magnified in the lower panels. The lower left panel shows iron in the anterior midgut. The lower right panel shows the iron region in the middle midgut. The X-ray absorption spectra shown in Fig. 3 labeled “gut–FAC” are from this iron region. **B** The anterior midgut of an intestine dissected out of a larva raised on 250 µM BPS is shown in the upper panel to demonstrate the proposed region of ferric reductase activity and hence the site of intestinal iron absorption. Asterisks denote the characteristic pink color precipitate in the animals that consumed BPS-supplemented diet. In the lower panel, the X-ray fluorescence image of iron verifies that iron is readily observable at the site of the pink BPS–Fe^2+^ precipitate in a small distinct region of the anterior midgut in BPS-treated larvae. X-ray absorption spectra in Fig. 3 labeled “gut–BPS” are from this region. Color scale depicts photon counts per pixel

The pink precipitate appeared to be associated with cellular membranes and we therefore hypothesized that it identified cells with extracellular, membrane-bound ferric reductase activity, as previously described in the mammalian iron uptake system and predicted for the insect iron uptake system. According to this hypothesis, ferric reductase activity would locally convert iron into ferrous form to enter the intestinal epithelial cells through Malvolio and thus, cells harboring such activity define the site of intestinal iron absorption. In BPS-treated animals, three chelator molecules should bind tightly to the divalent iron [63–65], forming a membrane-impermeable complex and thus blocking organismal iron absorption, explaining the iron deficiency phenotype associated with this treatment. To test this hypothesis, freshly dissected intestines from BPS- and FAC-treated individuals were placed on coverslips, allowed to air-dry in ambient conditions and analyzed by X-ray absorption imaging at beamline 2–3 at SSRL (Fig. 2).

First, we verified that the distribution of iron in the intestines dissected from FAC-treated larvae followed the pattern observed with the Prussian blue staining (compare Fig. 2A with Fig. 1A). Next, we visualized iron in the intestines dissected from BPS-treated larvae (Fig. 2B). Significant concentrations of iron were detected in the same position where the pink precipitate appears, suggesting that the pink precipitate represents accumulation of the expected BPS–iron complex.

**Fig. 3:**
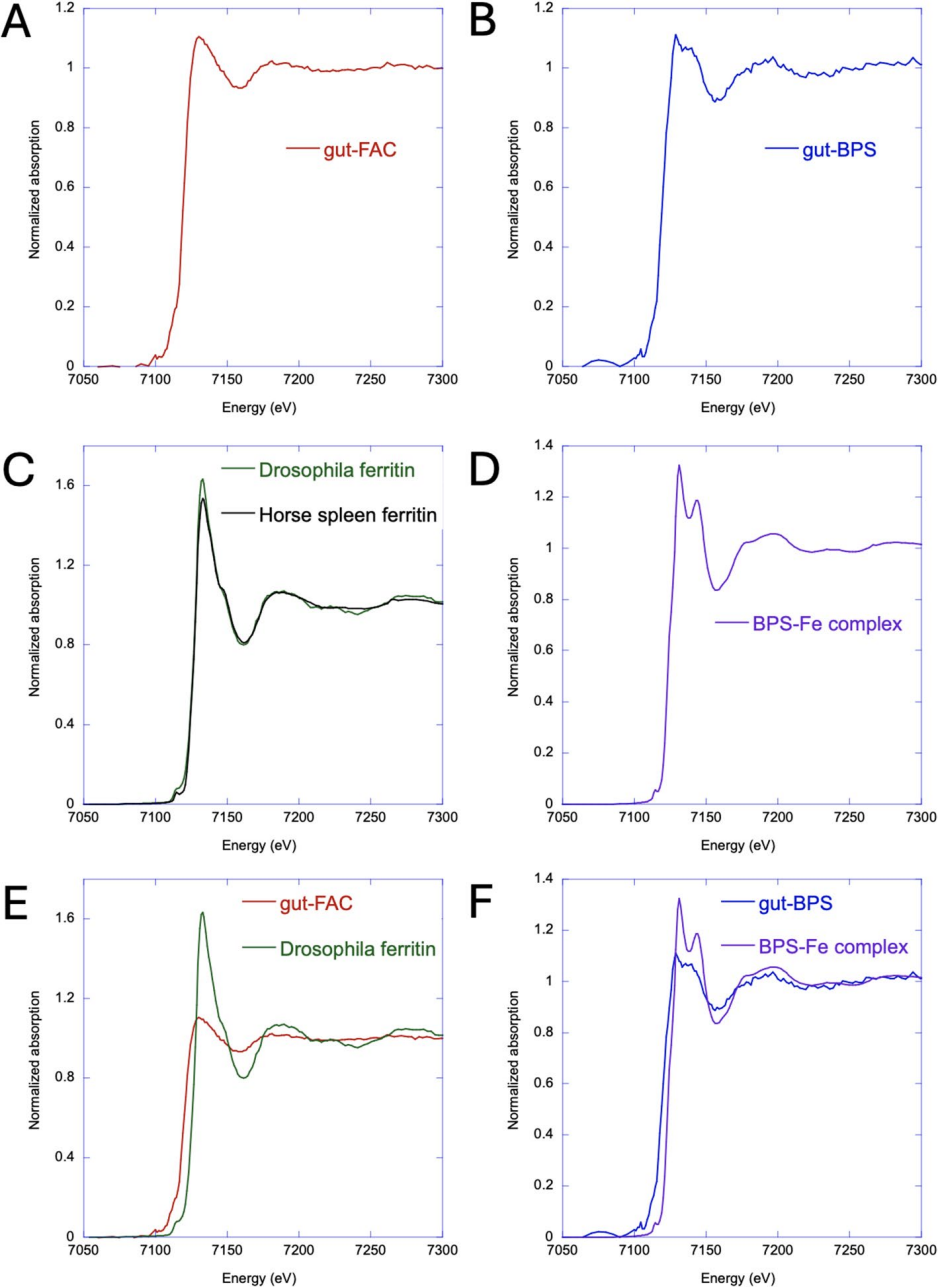
XANES spectra obtained from areas of interest in the air-dried intestines of *Drosophila melanogaster* 3rd instar larvae and of purified ferritin and a BPS–iron complex synthesized from ferrous iron but under aerobic conditions. **A** Spectrum obtained from the iron region of the larva shown in Fig. 2A raised on 1 mM FAC. **B** Spectrum obtained from the proposed site of iron absorption in the anterior midgut of the intestine shown in Fig. 2B dissected out of a larva raised on 250 µM BPS (pink color precipitates). The above XANES spectra were recorded at the SSLR synchrotron. **C** Purified ferritin from *Drosophila melanogaster* adult flies raised on 1 mM FAC was compared to commercially available horse spleen ferritin. **D** Spectrum of a 3:1 BPS to ferrous sulfate mixture. The XANES spectra in C and D were recorded at the Soleil synchrotron. **E** Comparison between the *Drosophila* ferritin and intestinal iron region spectra. **F** Comparison of the BPS–Fe^2+^ complex to the intestinal site of iron absorption spectra. Note that the energy shifts observed in panels E and F are meaningless since both series of XANES spectra were recorded at two beamlines with different energy calibration conditions. A comparison of spectral derivatives of normalized XANES spectra in this figure, an overlay of the XANES spectra shown in panels A and B, and an overlay of reference aqueous spectra for Fe^2+^ and Fe^3+^ ions are also provided (Fig. S2, SI)

To probe the nature of the species detected in the iron region of the FAC-treated animals (gut–FAC) and those giving rise to the pink color in the newly defined iron absorption region of BPS-treated animals (gut–BPS), we obtained XAS data in the XANES mode from these two areas at beamline 2–3 at SSRL (Fig. 3A, B). The two samples have distinct features, reflecting iron species with different coordination spheres (notably the gut–BPS spectrum is noisier because of the Fe depletion). Specifically, the gut–BPS spectrum displays an extra feature at around 7140 eV. These spectra were compared to those of the synthesized BPS–Fe(II) complex, commercially available horse spleen ferritin and *Drosophila* ferritin purified from adult flies raised on a 1 mM FAC diet. XANES spectra of these standards were collected at the MARS beamline of the SOLEIL synchrotron (Fig. 3C, D).

The spectra of the insect and horse spleen ferritins are quite similar (Fig. 3C), consistent with both having predominantly Fe(III) sites in the ferritin iron core (Fe_2_O_3_ oxide form) but also on the ferritin shell with coordination to mainly oxygen atoms, with potentially few Fe(II) atoms present in the ferroxidase centers of the protein shell [70–73]. In contrast, the BPS–iron complex displays an octahedral coordination to nitrogen atoms [63–65] and its XANES spectrum (Fig. 3D) is quite different from those of ferritin, showing a pronounced feature at around 7140 eV, similar to that observed in the gut–BPS sample (Fig. 3B). Indeed, the spectra for gut–BPS and the BPS–iron complex (Fig. 3F) are quite similar, strongly suggesting that the iron species present in the pink precipitate region of BPS-treated animals corresponds mostly to the BPS–iron complex, in agreement with the observations on the images of Fig. 2. Comparison of the XANES spectra for gut–FAC with that of *Drosophila* ferritin (Fig. 3E) does not yield a clear conclusion regarding the nature of the iron species in this region; while it is likely that ferritin contributes to the signal of the iron region in the FAC-fed animals, there might be other iron species that also contribute to the spectrum.

**Fig. 4:**
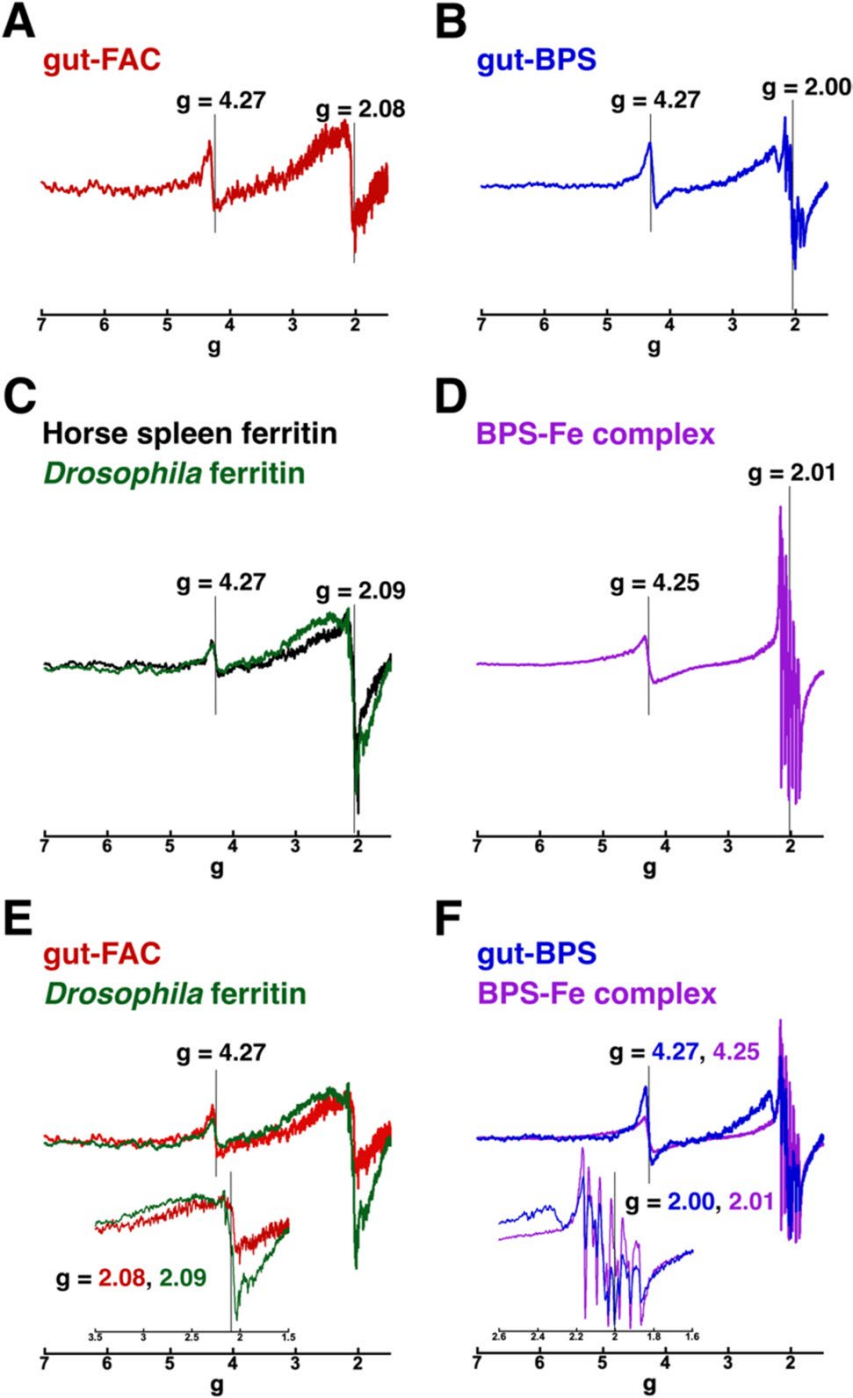
Normalized EPR spectra obtained from larval intestines, purified ferritins and a chemically synthetized BPS-iron complex. **A** Normalized EPR spectrum obtained from intestines dissected out of wandering 3rd instar larvae raised on 1 mM FAC (gut–FAC). Characteristic EPR signals appear at *g* values 4.27 and 2.08. The relative intensity between these two signals (*g* ~ 2 and *g* = 4.27) is the same in A and B, but no conclusion can be drawn regarding the concentration of the high-spin ferric species that gives rise to the isotropic signal at *g* = 4.27 (Fig. S5, SI). **B** Normalized EPR spectrum obtained from intestines dissected out of wandering 3rd instar larvae raised on 250 µM BPS (gut–BPS). While the first signal remains unchanged, a new multiple line signal at *g* = 2.00 is observed, that can be ascribed to the presence of a small amount of aqueous Mn^2+^ species (Fig. S3A, SI). **C** Normalized EPR spectra of *Drosophila* and horse spleen ferritins significantly overlap and show identical *g* values.** D** EPR spectrum of a 3:1 chemically synthetized BPS-iron complex prepared aerobically. The spectrum shows a signal at g value 4.25 and a multiple line signal at *g* = 2.01, that can be deconvoluted as a mixture of aqueous Mn^2+^ and BPS-Mn^2+^ complex (Fig. S3B, SI). **E** Comparison between EPR spectra from *Drosophila* ferritin and the iron-fed larval intestines. **F** Comparison of EPR spectra from BPS-iron complex and BPS-fed larval intestines

To gain further insight into the iron speciation at the examined regions of FAC-fed (gut–FAC) versus BPS-fed (gut–BPS) animals, samples of freshly dissected intestines from wandering larvae raised on 1 mM FAC (Fig. 4A) or 250 µM BPS (Fig. 4B) were analyzed by X-band EPR spectroscopy. Both samples displayed a similar feature at *g* = 4.27, but distinct signals at *g* ~ 2: while gut–FAC shows a broad signal at *g* = 2.08, the gut–BPS sample displays a multiple signal centered at *g* = 2.00. These EPR spectra were compared to those collected for synthesized BPS–Fe(II) complex, commercially available horse spleen ferritin and purified *Drosophila* ferritin (Fig. 4C, D). The EPR spectra of the two ferritins are quite similar (Fig. 4C) and exhibit two features: a broad signal around *g* = 2.09, attributed at the ferritin core, as previously reported for horse spleen ferritin [74]; and a signal at *g* = 4.27 that has been associated with the protein shell to solitary high-spin Fe^3+^ ions bound to apoferritin [75]. These results suggest a very similar structure of the iron core in *Drosophila* ferritin, as compared to horse spleen ferritin, consistent with what was observed by XANES analysis above. On the other hand, the EPR spectra from *Drosophila* ferritin and the iron-fed larval intestines (Fig. 4E) are very similar, supporting the notion that ferritin contributes to the iron speciation in intestines of FAC-fed animals. In contrast, the EPR spectrum of the synthesized BPS–Fe complex (3:1) (Fig. 4D) is quite different from those of ferritin; it shows a signal at *g* = 4.25 (probably due to a small amount of high-spin Fe^3+^ species) and a multiple line signal at *g* = 2.01 that can be ascribed to the presence of a small amount of aqueous Mn^2+^ species (Fig. S3A, SI), a common contamination in iron salts [76, 77]. Indeed, we also checked our own ferrous sulfate salt and found it contained 0.3% manganese (Fig. S4A, SI). A comparison of the EPR spectra of gut–BPS with that of the BPS-iron complex shows striking similarities (Fig. 4F); the BPS-fed larval intestines also display a Mn^2+^ signal that can be deconvoluted as a mixture of aqueous Mn^2+^ and BPS-Mn^2+^ complex (Fig. S3B, SI). The fact that these Mn^2+^ species were not observed in the gut–FAC samples probably reflects an altered manganese speciation in the BPS-fed animals. ICP-OES analysis of whole larvae indicated an increase in total manganese content in BPS-treated larvae (Fig. S1B). We also checked whether our BPS reagent was free of metal contaminants (Fig. S4B–D, SI). The results showed that the increased manganese observed in larvae fed with BPS (by 45% compared to control animals) cannot be explained by a trace amount (7 parts per billion) of manganese in the reagent and merits further consideration in future studies. Last, it should be noted that the detection of Fe^2+^ species by EPR is limited due to their nature and the experimental conditions used here (Fig. S6, SI) [78, 79].

In conclusion, our EPR data together with XANES qualitative analysis support the notion that *Drosophila* ferritin contributes to the iron pool in the intestines of FAC-fed animals, while the iron species that yields the pink coloration in intestines of BPS-fed larvae corresponds to a BPS-iron complex. The formation of this complex in a specific iron region adjacent to the anterior-most part of the midgut suggests that it may be a site of iron absorption in this insect.

## Discussion

The present study forms part of our recent efforts to combine spectroscopies that were initially developed to study metalloorganic complexes in solution with microscopical techniques and whole tissue samples [80–82]. This approach opens the field of cell biology to chemical investigations in situ*.* Direct imaging of metal ions through X-ray fluorescence imaging is therefore enhanced with detailed understanding of the chemical environment and speciation of the accumulated metal ions within the biological milieu. Here, the technique of monitoring the colorimetric properties of BPS–Ferrous iron complexation to label membrane ferric reductase activity [60, 61] was applied to the *D. melanogaster* intestine. In this way, we identified a relatively narrow ferric iron reductase region at the “neck” of the anterior midgut, immediately adjacent to the region that turns on ferritin transcription and translation upon exposure to iron [32, 53, 55, 69]. While the identity of the insect ferric reductase responsible for this activity remains unknown [22], the assay we have developed could prove useful to probe candidate DcytB homologs in *D. melanogaster* [52].

We infer that the newly identified region is the site of dietary iron absorption, based on existing knowledge regarding the conserved role of Malvolio (*Drosophila* DMT-1) in iron uptake [31] and the role of DcytB in this process from studies in rodents [41]. The rather restricted domain where BPS-iron complexes were observed raises the question of whether the Malvolio expression pattern is similarly narrow [83]. If ferrous iron reduction takes place in the newly identified region, the cells that apparently absorb iron in the insect are neighboring cells to those that accumulate the metal when iron is present in the diet at high concentrations, suggesting further transport mechanisms that require clarification. One known export protein for iron is Zip13 [84]. The question of whether ferritin itself may transport the iron or whether *Drosophila* transferrin [85–90] may have such a role remains to be investigated more closely.

A puzzling observation in the literature that still calls for an explanation is the genetic interaction observed between transferrin and ferritin, whereby intestinal RNA interference of either *Tsf1* or *Fer1HCH* results in severe phenotypes, which are not seen when both genes are silenced simultaneously [85], despite both proteins being implicated in iron trafficking between cells [85–90]. Genetic interaction studies may, however, be confounded by the activity of genes in different regions. Our study’s contribution to the growing complexity of this system (reviewed in [20, 22]) is precisely the documentation of an additional cell type and region of interest where ferric reductase activity produces ferrous iron, which is presumably transferred intracellularly through Malvolio. Future studies should aim to uncover the identity of the ferric reductases and map their expression along the intestinal epithelium.

Importantly, we have demonstrated through two spectroscopic techniques, XANES and EPR, that the pink precipitates observed are indeed BPS–Fe complexes with a small component of manganese contamination. In both cases, data analysis focused on unique aspects of the spectra that were observed from standard chemical preparations and tissue samples. The tissue spectra from FAC and BPS fed larvae showed characteristic features, which could be carefully and unambiguously attributed to the components expected from the dietary manipulation. Thus, an important biological problem—the site of iron absorption in an insect epithelium—was determined with the support of chemical spectroscopies.

## Supplementary Information

Below is the link to the electronic supplementary material.

Supplementary file1 (PDF 1458 KB)

## Data Availability

All the data presented in this manuscript, including the original spectroscopic measurement files, are available upon request.
