# Supplementary material for: In situ detection of ferric reductase activity in the intestinal lumen of an insect
Source: J Biol Inorg Chem. 2024 Dec 1;29(7-8):773–84. doi: 10.1007/s00775-024-02080-y (PMC11638316; doi:10.1007/s00775-024-02080-y)
Supplement: Supplementary file 1 — Supplementary file1 (PDF 1458 KB) [file 775_2024_2080_MOESM1_ESM.pdf]

Supplementary Information for "*In situ* detection of ferric reductase activity in the intestinal lumen of an insect" by Anna Karen Hernández-Gallardo et al. (JBIC, 2024)

Analysis of total iron content in larvae grown with 250 µM BPS or 1 mM FAC verified that the treatments were effective as expected (Fig. S1A). The EPR analysis of BPS-treated larval intestines revealed a conspicuous Mn^{2+} signal (Fig. 3). An unexpected increase in total manganese content was also observed under BPS feeding (Fig. S1B).

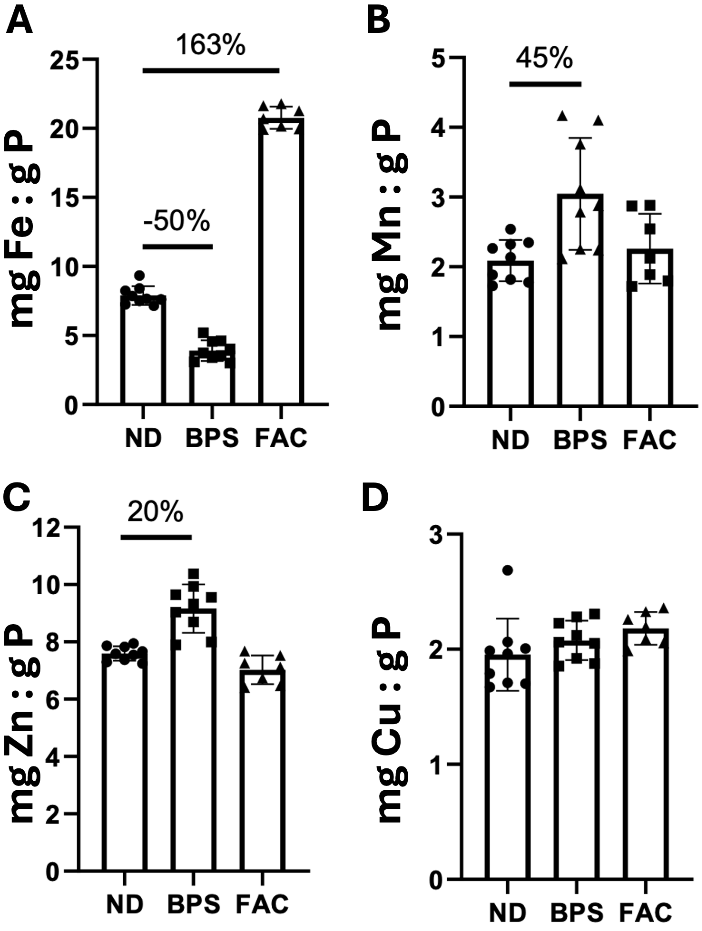

Fig. S1 Whole body wandering 3rd instar larva metal determinations by ICP-OES. **A** As expected, larvae raised on a diet containing 250 µM BPS (squares) show significant less (-50%) body accumulation of iron compared to those raised on normal diet and larvae raised on a diet containing 1 mM FAC (triangles) show significant more (+163%) body accumulation of iron compared to control animals. **B** Unexpectedly, larvae raised on a diet containing 250 µM BPS (squares) show significant more (+45%) body accumulation of manganese compared to those raised on normal diet and **C** significant more (+20%) body accumulation of zinc. **D** Body accumulation of copper was unaffected by the treatments.

XANES spectra were acquired in two different facilities (SSRL and Soleil), under distinct calibration conditions, which complicates direct comparisons between the results obtained from tissue samples and chemical standards. Further details of spectral comparisons from the results presented on the paper's Fig. 3 are provided below (Fig. S2).

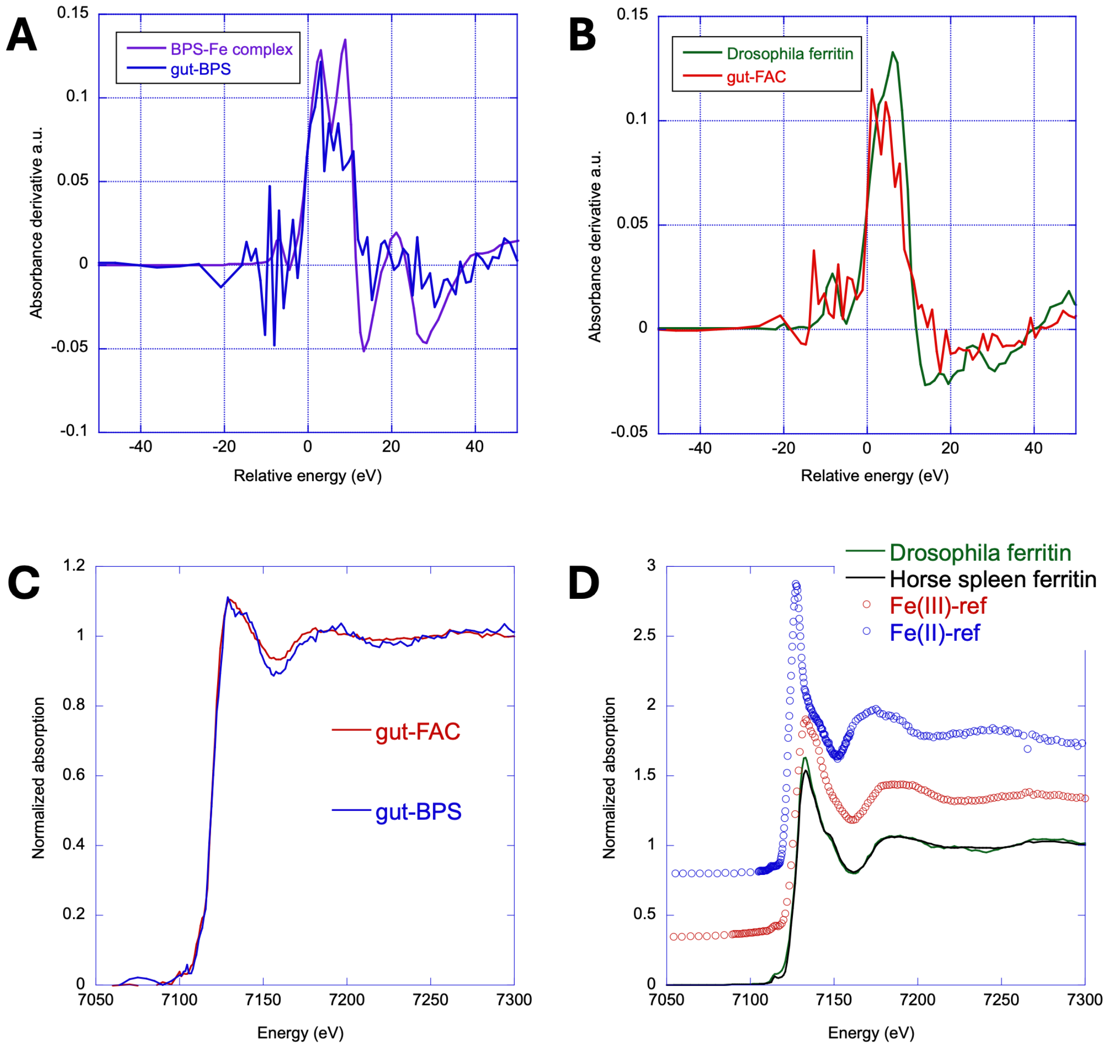

Fig. S2 Further analysis of the XANES spectra presented in Fig. 3. **A** Comparison between BPS-Fe complex and gut-BPS spectra using the first derivative of the XANES spectra, after the spectra have been realigned in relative energy scale. **B** Similar comparison between *Drosophila* ferritin and gut-FAC. **C** XANES spectra obtained from areas of interest in the air-dried intestines of *Drosophila melanogaster* 3rd instar larvae (merge of Fig. 3 A, B). The gut-FAC spectrum was obtained from the iron region of the larva raised on 1 mM FAC, from the anterior midgut of the intestine where dietary-supplemented iron accumulates (see Fig. 1A, 2A). The gut-BPS spectrum was obtained directly from the pink precipitates in a separately treated larva raised on 250 µM BPS (Fig. 1B, 2B). Note a characteristic shoulder in the gut-BPS spectrum that is turned down in the gut-FAC spectrum. **D** XANES spectra of purified ferritin from *Drosophila melanogaster* adult flies raised on 1 mM FAC and commercially available horse spleen ferritin are compared with an Fe(II) reference (aquo Fe(II) in acidic reducing conditions) and an Fe(III) reference (aquo Fe(III) in acidic conditions) [72].

In the EPR spectra, the presence of a manganese contaminant in the BPS-iron complex synthesized and used in this study was detected, and this trace contaminant could also be detected in the intestinal sample from BPS-treated larvae (Fig. S3). ICP-OES analysis confirmed the presence of manganese in the BPS-iron complex and trace amounts of manganese on the pure BPS as purchased from Sigma (Fig. S4).

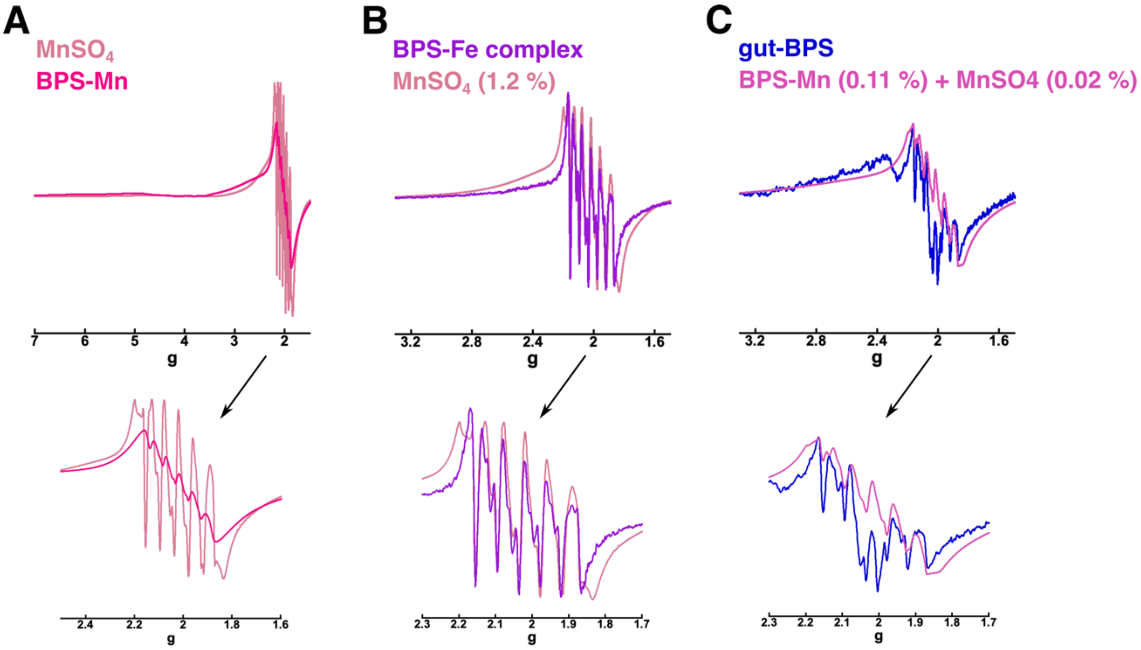

Fig. S3 EPR spectra obtained from aqueous $MnSO_4$, BPS-manganese complex and their comparison with BPS-iron complex and BPS-fed larval intestines. **A** EPR spectrum obtained from $MnSO_4$ in HEPES buffer with 50 % glycerol and its comparison with EPR spectrum of a 3:1 BPS to manganese sulfate mixture (450 mM, 150 mM) prepared aerobically. At g ~2, the spectra show the six signals associated to hyperfine coupling with the nuclear spin of Mn^{2+} (d^5, S=5/2, I=5/2). **B** The signals around g = 2 observed for the BPS-Fe complex can be reproduced with 1.2 % of the $MnSO_4$ spectrum. **C** The signals around g = 2 displayed by the gut-BPS sample can be partially reproduced with a combination of 0.11 % of the BPS-Mn and 0.02 % of $MnSO_4$ spectra.

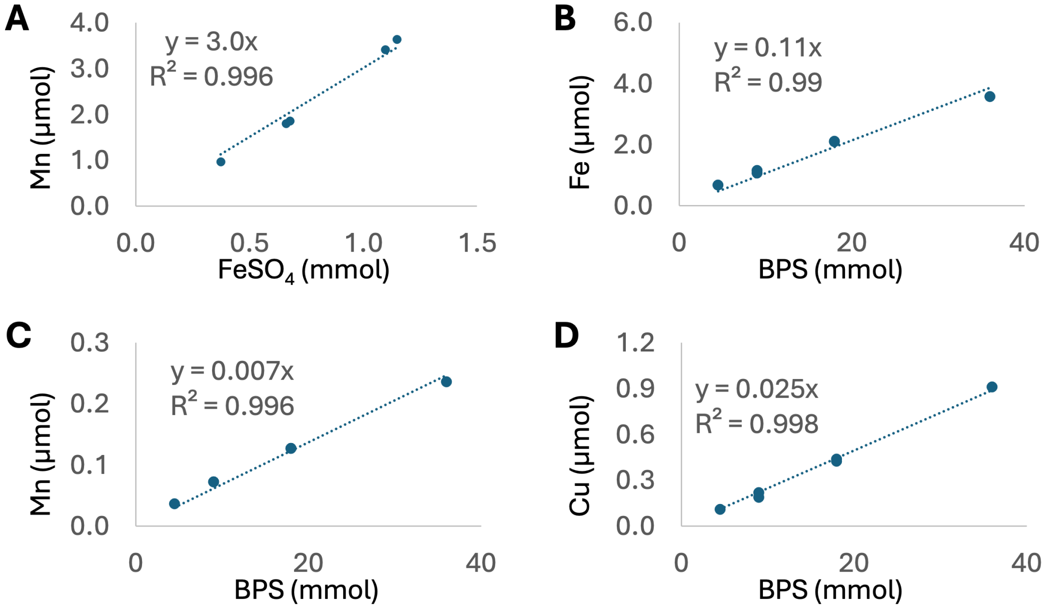

Fig. S4 ICP-OES measurements of reagents used in this study for the preparation of the BPS-Fe complex. **A** For every 1000 iron atoms detected in the ferrous sulfate salt we detect 3 manganese atoms, meaning that there is a 0.3% contamination of the salt. The plot shows measurements of iron in the X-axis and of manganese in the Y-axis. Note the difference in units (mmol vs µmol, respectively). **B** BPS as purchased from SIGMA was analyzed in water and we identified 0.1 ppm Fe, **C** 7 ppb Mn, and **D** 25 ppb Cu attached to the compound. Here ICP-OES data are only on the Y axis, while on the X-axis we plot the concentration of BPS used.

We now turn our attention to the EPR signal associated to high spin ferric species (g=4.27). The nature of the g=4.27 signal in the high-spin Fe^{3+} (S = 5/2) species (Fig. S5A) arises in systems with a symmetry less than octahedral or tetrahedral; in these, the spin Hamiltonian have a term called zero field splitting (ZFS) that depends on two factors D and E (Eq. 1).

$$\mathcal{H}_{ZFS} = D\left[S_z^2 - \tfrac{1}{3}S(S+1)\right] + E(S_x^2 - S_y^2) \text{ .... Eq. 1}$$

Where, D is the axial ZFS parameter (for z ≠ x = y), and E is rhombic ZFS parameter (for x ≠ y) and the E/D ratio (λ) is ≥ 1/3. These terms split the (2S + 1) m_S degeneracy of the ground state in the absence of a magnetic field (Figure S5B).

In the case of large rhombic distortion (Fig. S5C), the ZFS energy diagram presents three equally spaced doublets splits in energy. The g = 4.3 signal derives from the middle doublet and is independent of orientation, that is, $g_x = g_y = g_z = 4.3$. The isotropic nature of this signal allows it to be easily observed and appears about three orders of magnitude taller than the g = 9.7 signal. Thus, this signal is observed even when only minute quantities of high-spin ferric (with large rhombic distortions) species are present. Because of this, very small changes in the amount of the high-spin (rhombic) Fe^{3+} species can lead to a significant change in signal intensity, without necessarily representing a significant change in the concentration of those species.

In the gut-FAC (Fig 4A in the manuscript), the g=4.27 signal is attributed to the protein shell with high-spin Fe^{3+} ions bound to apoferritin, as previously reported [75]; this signal is similar to that of horse spleen ferritin and *Drosophila* ferritin samples (Fig. 4C in the manuscript). In contrast, in gut-BPS (Fig 4B in the manuscript), the g=4.27 signal is associated to unidentified high-spin Fe^{3+} species. In both cases, it is not possible to determine the concentration of those species for the reasons given above.

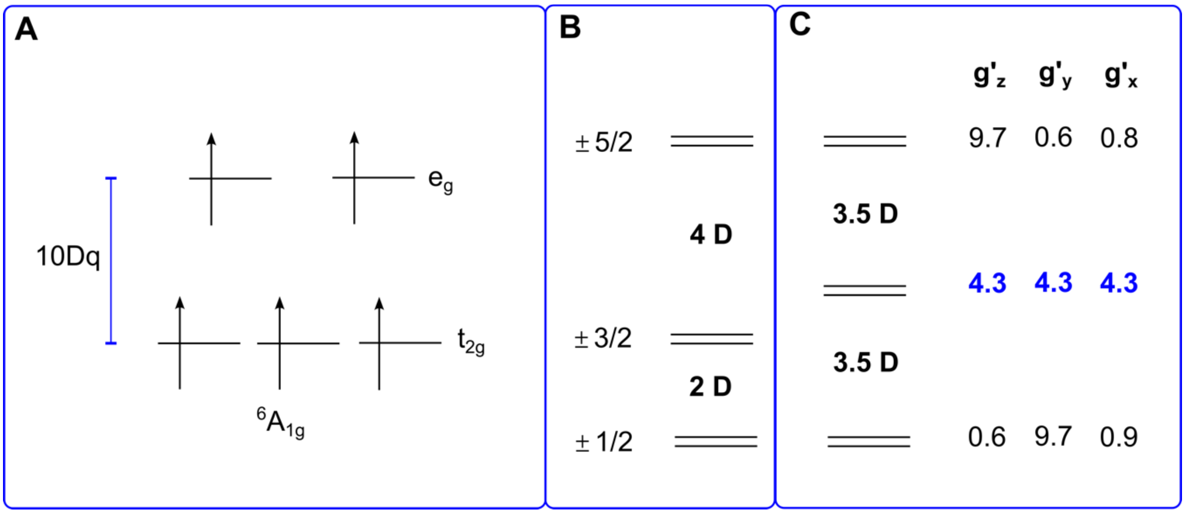

Fig. S5 One electron d orbital energy level diagram in an octahedral ligand field (A), splitting of the m_S levels with axial (B) and large rhombic distortion (C) at zero field with g' values listed (C) of a high spin ferric (S = 5/2). *Figure adapted from Inorganic electronic structure and spectroscopy, 1999, Vol. II; edited by Edward I. Solomon and A. B. P. Lever., pp. 40* [79].

EPR of high spin ferrous species

Fe^{2+} species have a d^6 configuration and can be low-spin or high-spin ferrous centers (Fig. S6A, B). The low-spin ferrous center has a S = 0, which is silent in EPR. The high-spin ferrous center has a S = 2 with m_S = ± 2, ± 1, 0. Even in the absence of magnetic field, the m_S = ± 2 and m_S = ± 1 spin sublevels split in energy; this phenomenon is called zero field splitting (ZFS) and it can be different for axial or rhombic symmetries. In Fig. S6C, D, the splitting of the m_S sublevels in a ferrous center with axial symmetry are shown, while Fig. S6E shows the splitting in a rhombic symmetry. In the latter case, integer spin EPR

signals can be observed at low field (X-band) and low temperature (4 K), whether the EPR experiment is performed with the microwave field parallel or perpendicular (conventional mode) to the magnetic field. Hence, X-band (9.1 GHz) EPR spectra of ferrous systems have been reported where integer spin signals appear at fields < 100 mT and g values range from 9-16 [78]. In turn, due to the very large zero-field splitting, only at high magnetic fields can EPR signals associated to $\Delta\, m_S = \pm 1$ be observed (namely the $m_S = 0$ and one of the $m_S \pm 1$ levels or between one of the $m_S \pm 2$ levels and one of the $m_S \pm 1$ levels). The high magnetic field required is often beyond the reach of common instruments, hence with a standard X-band (9.5 GHz) spectrometer setup, which typically has an electromagnet that ramps the field up to 1.2 T, is not possible to reach the resonance condition for the transitions mentioned above. However, with home-built spectrometers with a magnet that covers the magnetic field range of 5.2 to 6.6 T, and a set up for low temperatures (5 K) and very high frequencies (170, 222.4 and 331.2 GHz), it is possible to obtain the spin-Hamiltonian parameters of ferrous systems [77]. Due to the characteristics of high-spin ferrous centers, alternative approaches are often used to study high-spin ferrous sites experimentally, such as magnetic susceptibility, variable temperature, variable field magnetic circular dichroism (VTVH-MCD) or XAS (see Bioinorganic Spectroscopy in [79]).

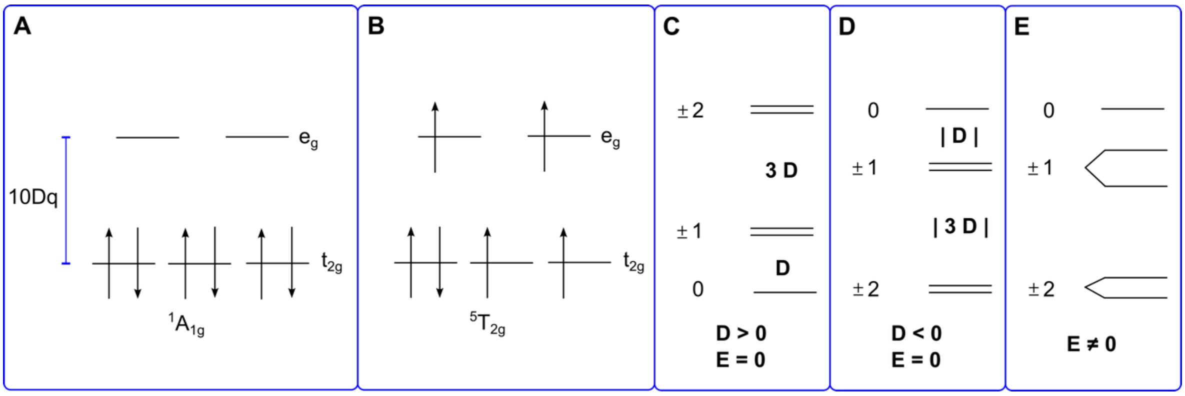

Fig. S6 One electron d orbital energy level diagrams for low-spin (A) and high-spin (B) ferrous center in an octahedral ligand field. Zero field splitting diagrams for a high-spin ferreous center with D > 0 and E = 0 (C), D < 0 and E = 0 (D) and E ≠ 0 (E). D and E are two factors in the spin Hamiltonian term called zero field splitting (ZFS) as follows: $\mathcal{H}_{ZFS} = D\left[S_z^2 - \frac{1}{3}S(S+1)\right] + E(S_x^2 - S_y^2)$; where D is the axial ZFS parameter (for z ≠ x = y) and E is rhombic ZFS parameter (for x ≠ y).
